# Supplementary material for: Supercritical Carbon Dioxide Extraction of Four Medicinal Mediterranean Plants: Investigation of Chemical Composition and Antioxidant Activity
Source: Molecules. 2021 Sep 20;26(18):5697. doi: 10.3390/molecules26185697 (PMC8468049; doi:10.3390/molecules26185697)
Supplement: Supplementary file 1 [file molecules-26-05697-s001.zip › molecules-1377279-supplementary.pdf]

Supplementary Materials

Supercritical Carbon Dioxide Extraction of Four Medicinal Mediterranean Plants: Investigation of Chemical Composition and Antioxidant Activity

Lara Čižmek [1,2], Mojca Bavcon Kralj [3], Rozelindra Čož-Rakovac [1,2], Dmitrii Mazur [4,5], Nikolay Ul'yanovskii [5],
Marko Likon [6] and Polonca Trebše [3,*]

[1] Laboratory for Aquaculture Biotechnology, Division of Materials Chemistry, Ruđer Bošković Institute, Bijenička 54, 10000 Zagreb, Croatia; lcizmek@irb.hr (L.Č.); rrakovac@irb.hr (R.Č.-R.)
[2] Center of Excellence for Marine Bioprospecting (BioProCro), Ruđer Bošković Institute, Bijenička 54, 10000 Zagreb, Croatia
[3] Faculty of Health Sciences, University of Ljubljana, Zdravstvena pot 5, 1000 Ljubljana, Slovenia; mojca.kralj@zf.uni-lj.si
[4] Chemistry Department, Lomonosov Moscow State University, Leninskie Gory 1/3, 119991 Moscow, Russia; neodmitrii@gmail.com
[5] Core Facility Center "Arktika", Lomonosov Northern (Arctic) Federal University, nab. Severnoy Dviny 17, 163002 Arkhangelsk, Russia; n.ulyanovsky@narfu.ru
[6] Škrlj, d.o.o., Batuje 90, 5262 Črniče, Slovenia; marko.likon@telemach.net
* Correspondence: polonca.trebse@zf.uni-lj.si; Tel.: +386-1-300-11-15

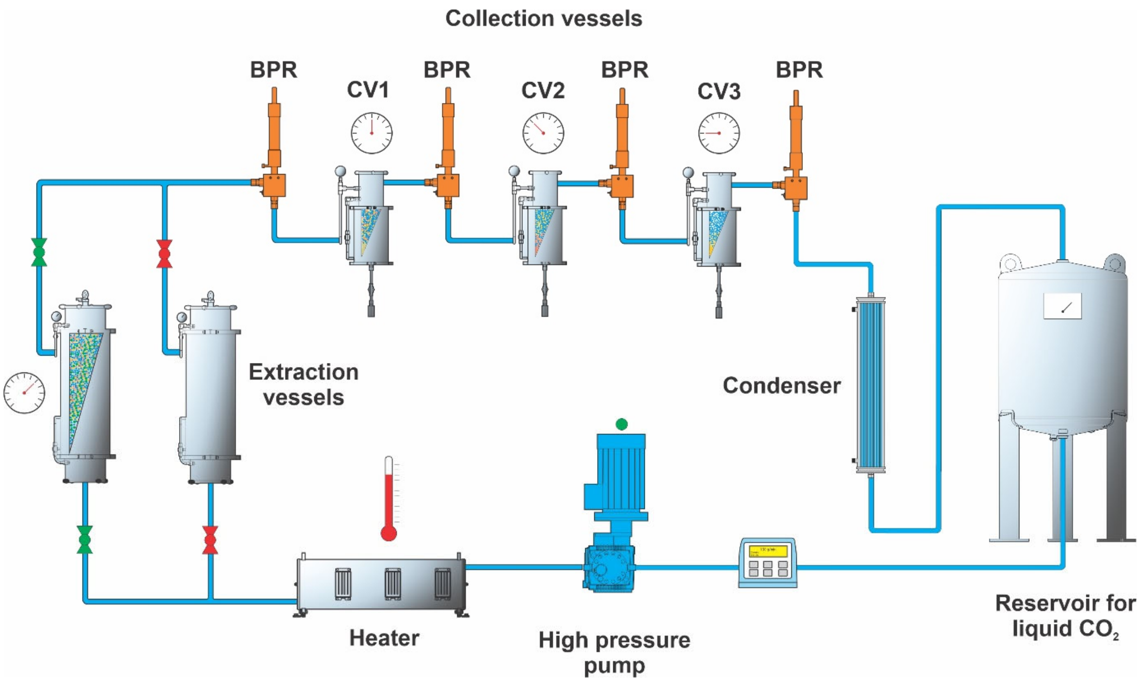

Figure S1. Schematic diagram of the supercritical extraction system.

Table S1. LC-MS (negative mode) analysis of plant extracts (RDB - rings and double bonds equivalent; RT – retention time).

Formula	RDB	Compound	RT	Chamomile	Yarrow	St. John's wort	Curry plant	Comments/possible candidates
C16H20O8	7	Hydroxycinnamic acid glucoside?	6.27		+			
C10H14O3	4	Some polyphenol	7.30				+	
C13H18O4	5	Some polyphenol	7.58			+	+	
C24H30O9	10	Some polyphenol	7.61				+	
C21H30O10	7	Diglucoside?	7.62		+			
C11H16O3	4	Some polyphenol	7.96			+		
C11H16O3	4	Some polyphenol	7.97				+	Psoralen
C18H34O5	2	Trihydroxyoctadecenoic acid	7.98	+				
C25H32O9	10	Some polyphenol	8.01				+	
C19H24O8	8	Functionalized polyphenol	8.11		+			
C14H20O4	5	Functionalized polyphenol	8.23			+	+	
C19H24O8	8	Glucosilated coumarin?	8.26		+			
C25H24O3	14		8.43	+				
C19H26O8	7		8.48		+			
C18H16O8	11	Hydroxy-methoxy-flavone	8.87		+			
C21H28O5	8	Functionalysed phytosterol	9.33				+	
C13H16O2	6	Butyl cinnamate	9.34				+	
C18H30O4	4	Epoxy-hyroxy-octadecadienoic acid	9.54		+			
C28H30O5	14	Functionalized polyphenol	9.64	+				
C19H18O8	11	Dihydroxy-tetramethoxyflavone	9.73	+				5,6-Dihydroxy-7,8,3',4'-tetramethoxyflavone
C18H32O4	3	Epoxy-hyroxy-octadecenoic acid	9.74		+			
C17H26O4	5		9.86	+				
C18H32O4	3	Dihydroxy-or-epoxy-octadecadienoic acid	9.89	+	+			
C18H16O7	11	Dihydroxy-trimethoxyflavone	10.12		+			
C22H26O8	10	Functionalized polyphenol	10.16			+	+	Syringaresinol

Table S1. *Continued.*

Formula	RDB	Compound	RT	Chamomile	Yarrow	St. John's wort	Curry plant	Comments/possible candidates
$C_{23}H_{28}O_{8}$	10	Some polyphenol	10.51				+	
$C_{18}H_{32}O_{3}$	3	Hydroxyoctadecadienoic acid	10.60	+	+			
$C_{18}H_{34}O_{3}$	2	Hydroxyoctadecenoic acid	10.73	+				
$C_{18}H_{32}O_{4}$	3	Dihydroxyoctadecadienoic acid	10.83	+				
$C_{19}H_{30}O_{10}$	5		10.92				+	
$C_{22}H_{28}O_{8}$	9	Functionalized polyphenol	10.99				+	Lignan
$C_{24}H_{30}O_{8}$	10	Functionalized polyphenol	11.02				+	
$C_{18}H_{30}O_{3}$	4	Hydroxy-octadecatrienoic acid	11.05		+			
$C_{36}H_{64}O_{6}$	5		11.12	+				
$C_{20}H_{32}O_{5}$	5		11.12	+				
$C_{25}H_{32}O_{8}$	10	Functionalized polyphenol	11.29				+	
$C_{17}H_{20}O_{6}$	8	Helipyrone	11.31				+	
$C_{22}H_{30}O_{4}$	8		11.57		+			
$C_{18}H_{22}O_{6}$	8	Functionalized polyphenol	11.61				+	
$C_{24}H_{34}O_{4}$	8	Functionalized phytosterol	11.71			+		
$C_{22}H_{26}O_{8}$	10	Functionalized polyphenol	11.74			+	+	Syringaresinol isomer
$C_{22}H_{24}O_{7}$	11	Italipyrone	11.76				+	
$C_{29}H_{46}O_{6}$	7	Functionalized phytosterol	11.78			+		
$C_{21}H_{28}O_{4}$	8	Quinone derivative	11.78			+		
$C_{18}H_{30}O_{2}$	4	Octadecatrienoic acid	11.88	+	+	+		
$C_{24}H_{34}O_{3}$	8	Functionalized phytosterol	11.97				+	
$C_{23}H_{28}O_{8}$	10	Functionalized polyphenol	12.00				+	
$C_{25}H_{36}O_{4}$	8	Colupulone	12.02			+		
$C_{22}H_{26}O_{7}$	10	Functionalized polyphenol	12.13			+	+	Gmelinol? Clusin?
$C_{23}H_{26}O_{7}$	11	Methoxylated flavonoid	12.23				+	
$C_{18}H_{32}O_{2}$	3	Octadecadienoic acid	12.24	+	+			

Table S1. *Continued.*

Formula	RDB	Compound	RT	Chamomile	Yarrow	St. John's wort	Curry plant	Comments/possible candidates
C36H56O6	9	Prenylated phloroglucinol	12.26			+		
C23H28O7	10	Functionalized polyphenol	12.32				+	
C24H28O7	11	Functionalized polyphenol	12.44				+	
C16H32O2	1	Palmitic acid	12.50	+				
C38H56O8	11	Glyceride	12.50		+			
C35H52O5	10	Furohyperforin	12.61			+		
C24H30O7	10	Functionalized polyphenol	12.61				+	
C18H34O2	2	Octadecenoic acid	12.65	+	+			
C35H52O6	10	33-Hydroperoxyfurohyperforin	12.73			+		
C25H32O7	10	Functionalized polyphenol	12.77				+	
C29H42O4	9	Prenylated phloroglucinol	12.91			+		
C37H58O6	9	Prenylated phloroglucinol	13.04			+		
C27H34O7	11	Functionalized polyphenol	13.11				+	
C30H44O4	9	Colupone	13.12			+		
C35H50O5	11	Prenylated phloroglucinol	13.20			+		Pyrohyperforin or its isomer pyrano[7,28-b]hyperforin
C31H46O4	9	Adlupone	13.24			+		
C35H54O5	9	Prenylated phloroglucinol	13.35			+		
C32H48O4	9	Functionalized phytosterol	13.45			+		
C25H46O4	3	Octadecadienoic acid glyceride	13.50	+				
C35H52O5	10	8-Hydroxyhyperforin 8,1-hemiacetal	13.65			+		
C29H44O5	8	Functionalized phytosterol	13.69			+		
C35H52O4	10	Hyperforin	13.82			+		
C36H54O4	10	Adhyperforin	14.01			+		
C33H48O5	10	Prenylated phloroglucinol	14.04			+		
C35H52O5	10	Oxepahyperforin	14.70			+		

Legend: **Fatty acids or related** | **Functionalized phytosterol** | **Functionalized polyphenol** | **Prenylated phloroglucinols** | **Flavonoid**

Table S2. LC-MS (positive mode) analysis of plant extracts (RDB - rings and double bonds equivalent; RT – retention time).

Formula	RDB	Compound	RT	Chamomile	Yarrow	St. John's wort	Curry plant	Comments/possible candidates
C11H16O3	4	Functionalized polyphenol	6.25	+		+		psoralen
C8H10O3	4	Functionalized polyphenol	6.26				+	
C15H18O4	7	Sesquiterpenoid	6.72		+			
C15H26O4	3	Sesquiterpene	7.00	+				
C10H14O3	4	Functionalized polyphenol	7.25				+	
C13H14O3	7	Bitalin A	7.30				+	
C13H18O4	5	Functionalized polyphenol	7.55			+	+	
C15H20O4	6	Sesquiterpene	7.56	+				
C11H16O3	4	Functionalized polyphenol	7.95			+	+	
C10H8O3	7	7-Methoxycoumarin	8.00	+				
C14H20O4	5	Functionalized polyphenol	8.21			+	+	
C15H20O2	6	Sesquiterpenoid	8.34		+			
C15H18O3	7	Sesquiterpene	8.48	+				
C15H24O	4	Sesquiterpenoid	8.53			+		
C15H16O3+ C15H18O3	8	Sesquiterpene	8.62	+				
C11H16O2	4		8.63			+		
C17H20O5	8	Matricarin	8.78	+				
C15H20O4	6	Matricin	8.81	+				
C18H16O8	11	Trihydroxy-trimethoxyflavone	8.85		+			Rosmarinic acid
C13H12O2	8	Spiroether	8.86	+				
C15H24O3	4	Sesquiterpene	8.90	+				
C15H22O3	5	Sesquiterpenoid	9.02		+			
C15H18O2	7	Sesquiterpenoid	9.24		+			
C20H30O5	6	Sesquiterpenoid	9.24		+			
C15H16O4	8	Bitalin A acetate	9.24				+	
C21H28O5	8	Functionalized polyphenol	9.30				+	

Table S2. *Continued.*

Formula	RDB	Compound	RT	Chamomile	Yarrow	St. John's wort	Curry plant	Comments/possible candidates
C13H12O2	8		9.38	+				
C15H24O2	4	Sesquiterpenoid	9.39		+			
C28H30O5	14	Functionalized polyphenol	9.65	+				
C22H30O5	8	Functionalized polyphenol	9.7				+	
C19H18O8	11	Dihydroxy-tetramethoxyflavone	9.73	+				5,6-Dihydroxy-7,8,3',4'-tetramethoxyflavone
C16H18O4	8	Functionalized polyphenol	9.84				+	
C13H12O2	8	Spiroether	9.90	+				
C17H22O4	7	Sesquiterpenoid	10.11		+			
C18H16O7	11	Dihydroxy-trimethoxyflavone	10.12		+			5,4'-Dihydroxy-6,7,3'-trimethoxyflavone or 5,3'-Dihydroxy-6,7,4'-trimethoxyflavone or 5,6-Dihydroxy-4',7,8-trimethoxyflavone
C19H22O5	9	Functionalized polyphenol	10.13				+	
C22H26O8	10	Functionalized polyphenol	10.15				+	Syringaresinol
C17H24O4	6	Sesquiterpenoid	10.18		+			
C30H52O4	5	Functionalized phytosterol	10.20	+				
C18H28O2	5	Octadecatetraenoic acid	10.27		+			
C26H24O4	15	Functionalized polyphenol	10.33	+				
C17H20O4	8	Functionalized polyphenol	10.38				+	
C20H24O5	9	Functionalized polyphenol	10.57				+	Anhydro-secoisolariciresinol
C18H32O3	4	Hydroxyoctadecadienoic acid	10.61	+	+			
C15H22	5	Sesquiterpenoid	10.64			+		
C18H22O4	8	Functionalized polyphenol	10.83				+	
C14H14O2	8	Spiroether	10.93	+				
C22H32O5	7	Diterpenoid	11.01		+			
C18H30O3	4	Hydroxy-octadecatrienoic acid	11.04		+			

Table S2. *Continued.*

Formula	RDB	Compound	RT	Chamomile	Yarrow	St. John's wort	Curry plant	Comments/possible candidates
C15H26O	3	Bisabolol	11.20	+				
C15H24	4	Sesquiterpene	11.23		+			
C17H20O6	8	Functionalized polyphenol	11.30			+		
C17H20O6	8	Helipyrone	11.31				+	
C15H22	5	Sesquiterpene	11.34		+			
C15H22	5	Sesquiterpenoid	11.34			+		
C15H20O2	6		11.39		+			
C20H28O4	7		11.43		+			
C17H26O2	5	Sesquiterpenoid	11.55				+	
C18H22O6	8	Functionalized polyphenol	11.61				+	
C22H24O7	11	Italipyrone	11.75				+	
C29H40O6	10		11.86		+			
C18H30O2	4	Octadecatrienoic acid	11.89	+	+		+	
C19H24O6	8	Functionalized polyphenol	11.90				+	
C32H38O6	14		12.05		+			
C28H36O2	11	Functionalized phytosterol	12.07	+				
C22H26O7	10	Functionalized polyphenol	12.13				+	Gmelinol? Clusin?
C23H26O7	11	Functionalized polyphenol	12.23				+	
C18H32O2	3	Octadecadienoic acid	12.25	+	+			
C20H34O4	4		12.28				+	
C23H28O7	10	Functionalized polyphenol	12.31				+	
C24H28O7	11	Functionalized polyphenol	12.44				+	
C20H32	5	Diterpene	12.5		+			
C31H46O5	9	Functionalized phytosterol	12.63			+		
C25H30O7	11	Functionalized polyphenol	12.64				+	
C24H34O3	8		12.83				+	
C23H38O2	5		13.15			+		

Table S2. *Continued.*

Formula	RDB	Compound	RT	Chamomile	Yarrow	St. John's wort	Curry plant	Comments/possible candidates
C29H42O4	9	Coenzyme Q?	13.68			+		
C31H46O4	9	Adlupone	13.24			+		
C35H52O5	10	Prenylated phloroglucinol	13.38			+		
C26H44O7	5		13.44				+	
C36H54O5	10	Furoadhyperforin	13.57			+		
C29H42O4	9	Coenzyme Q?	13.68			+		
C35H52O4	10	Hyperforin	13.81			+		
C27H44O2	6	Prenylated phloroglucinol	13.88			+		
C36H54O4	10	Adhyperforin	13.99			+		
C28H46O2	6	Functionalized phytosterol	14.08			+		
C31H46O5	9	Functionalized phytosterol	14.13			+		
C35H52O5	10	Prenylated phloroglucinol	14.40			+		
C35H50O4	11	Prenylated phloroglucinol	14.55			+		
C34H54O3	9		14.62			+		
C30H44O5	9	Prenylated phloroglucinol	14.76			+		
C35H52O5	10	Prenylated phloroglucinol	14.77			+		
C35H52O4	10	Prenylated phloroglucinol	14.78			+		
C36H54O5	10	Prenylated phloroglucinol	14.96			+		

**Legend:**	**Fatty acids or related**	**Functionalized phytosterol**	**Functionalized polyphenol**	**Prenylated phloroglucinols**
	**Flavonoids**	**Sesquiterpenes, terpenoids**	**Coumarin**	
